# Pleural Fluid suPAR Levels Predict the Need for Invasive Management in Parapneumonic Effusions

**DOI:** 10.1164/rccm.201911-2169OC

**Published:** 2020-06-15

**Authors:** David T. Arnold, Fergus W. Hamilton, Karen T. Elvers, Stuart W. Frankland, Natalie Zahan-Evans, Sonia Patole, Andrew Medford, Rahul Bhatnagar, Nicholas A. Maskell

**Affiliations:** ^1^ Academic Respiratory Unit, University of Bristol, Bristol, United Kingdom; ^2^ North Bristol NHS Trust, Southmead Hospital, Bristol, United Kingdom; and; ^3^ Intensive Care Department, Musgrove Park Hospital, Taunton, United Kingdom

**Keywords:** empyema, pneumonia, pleural effusion, suPAR

## Abstract

**Rationale:** Parapneumonic effusions have a wide clinical spectrum. The majority settle with conservative management but some progress to complex collections requiring intervention. For decades, physicians have relied on pleural fluid pH to determine the need for chest tube drainage despite a lack of prospective validation and no ability to predict the requirement for fibrinolytics or thoracic surgery.

**Objectives:** To study the ability of suPAR (soluble urokinase plasminogen activator receptor), a potential biomarker of pleural fluid loculation, to predict the need for invasive management compared with conventional fluid biomarkers (pH, glucose, and lactate dehydrogenase) in parapneumonic effusions.

**Methods:** Patients presenting with pleural effusions were prospectively recruited to an observational study with biological samples stored at presentation. Pleural fluid and serum suPAR levels were measured using the suPARnostic double-monoclonal antibody sandwich ELISA on 93 patients with parapneumonic effusions and 47 control subjects (benign and malignant effusions).

**Measurements and Main Results:** Pleural suPAR levels were significantly higher in effusions that were loculated versus nonloculated parapneumonic effusions (median, 132 ng/ml vs. 22 ng/ml; *P* < 0.001). Pleural suPAR could more accurately predict the subsequent insertion of a chest tube with an area under the curve (AUC) of 0.93 (95% confidence interval, 0.89–0.98) compared with pleural pH (AUC 0.82; 95% confidence interval, 0.73–0.90). suPAR was superior to the combination of conventional pleural biomarkers (pH, glucose, and lactate dehydrogenase) when predicting the referral for intrapleural fibrinolysis or thoracic surgery (AUC 0.92 vs. 0.76).

**Conclusions:** Raised pleural suPAR was predictive of patients receiving more invasive management of parapneumonic effusions and added value to conventional biomarkers. These results need validation in a prospective multicenter trial.

**At a Glance Commentary**

**Scientific Knowledge on the Subject**

The ability to predict the clinical course of parapneumonic effusions would be invaluable to physicians when making management decisions at diagnosis. Currently, physicians rely on pleural fluid pH to determine the need for chest tube drainage despite a lack of prospective validation and no ability to predict the requirement for fibrinolytics or thoracic surgery.

**What This Study Adds to the Field**

In this prospectively collected cohort, a raised pleural suPAR (soluble urokinase plasminogen activator receptor) was highly predictive of patients who went on to receive more invasive management of parapneumonic effusions and added value to conventional biomarkers.

The clinical spectrum of pleural effusions related to infection is wide. From simple parapneumonic effusions that settle with conservative management through to grossly septated fibrinopurulent collections needing chest tube drainage, intrapleural fibrinolytics, or thoracic surgery for resolution. The process by which an effusion progresses down this cascade has been the subject of much research but the ability to predict which patients will require more aggressive intervention remains elusive (1). In 1980, Light and colleagues proposed the use of a pleural fluid pH cutoff of 7.2 to indicate the need for chest tube drainage on the basis that as bacteria metabolize and neutrophils phagocytose the pleural pH falls (2). This cutoff is referenced in the majority of international guidelines despite never being prospectively validated (3–5).

A defining feature in the spectrum of parapneumonic effusions is the dysregulation of the fibrinogenesis/fibrinolytic cascade and the subsequent development of loculations within the effusion. Loculations prevent adequate chest tube drainage, impede source control, can result in long-term respiratory compromise, and might even reduce the effectiveness of antibiotics (6). Pleural fluid pH, although a mainstay of initial management decisions, does not predict the development of loculations. A biomarker called suPAR (soluble urokinase plasminogen activator receptor) is theoretically a more appropriate guide for management. suPAR is the soluble form of uPAR (urokinase-type plasminogen activator receptor), which, once bound to endogenous uPA (urokinase), catalyzes the conversion of plasminogen to plasmin (a potent fibrinolytic). Originally documented in the plasma, serum, and urine of patients with HIV, pneumonia, sepsis, tuberculosis, and various solid tumors (7), more recent studies have shown suPAR also rises in infected ascitic and pleural fluid (8–11).

We aimed to assess the potential role of pleural fluid suPAR in the investigation and subsequent management of parapneumonic effusions using a prospectively collected cohort of patients.

Some of the results of this study have been previously reported in the form of an abstract (12).

## Methods

### Patients

Between 2009 and 2016, patients presenting to a UK tertiary pleural service with undiagnosed pleural effusions requiring a diagnostic thoracentesis were prospectively recruited to an observational study (IRAS ethics number 08/H0102/11). All had routine serum and pleural fluid analysis, including a full blood count, serum CRP (C-reactive protein), pleural fluid pH, glucose, and lactate dehydrogenase (LDH). At the time of pleural fluid sampling, pleural ultrasound was performed by a physician at least level 1 British Thoracic Society ultrasound-trained (or equivalent) with the presence of loculations documented. Repeat ultrasounds or computed tomography scans were performed if clinically indicated, and the development of loculations was recorded. Patients gave consent for storage of their baseline pleural fluid and serum samples in a −70°C freezer for future analysis.

Patients were followed up at 1 year to ascertain the final diagnosis of their pleural effusion, which was decided by two independent respiratory consultants based on prespecified criteria (*see* Appendix E1 in the online supplement). Patients were otherwise treated as per standard care; *see* Appendix E2 for local guidelines on parapneumonic effusion management.

### suPAR Testing

Pleural fluid and serum samples were analyzed from patients with an effusion secondary to infection. Those with frank pus on thoracentesis were excluded on the grounds that management for those cases is unequivocal. All patient samples were handled in accordance with a standardized study protocol; *see* Appendix E3 for full sample processing details and validation experiments of different sample preparation methods. suPAR levels were analyzed in duplicate (mean value presented) with high correlation observed (*R*^2^ > 0.99) using the suPARnostic AUTO Flex ELISA assay according to the manufacturer’s protocol (Virogates). This assay detects free suPAR (as well as domains II and III); it does not capture suPAR-scuPA (suPAR bound to single-chain urokinase) or suPAR-scuPA-PAI-1 (suPAR-scuPA bound to plasminogen activator inhibitor-1) complexes (13). As per protocol, samples were diluted until they fell within the workable range of the assay (0.2–15 ng/ml).

To explore suPAR levels in other etiologies, selected control subjects from the same cohort were also analyzed, including

1. transudative effusions secondary to congestive cardiac failure or hepatic hydrothorax,
2. nonloculated malignant effusions,
3. loculated malignant effusions, and
4. malignant effusions that were simple at baseline but became loculated at later time points receiving intrapleural fibrinolytics (urokinase).

### Statistical Analysis

Patient data are reported as the median/interquartile range (IQR)/range for continuous variables. The statistical differences between groups were analyzed using a nonparametric Mann-Whitney *U* test.

The correlation between serum/pleural suPAR and conventional biomarkers (including serum CRP and neutrophils, pleural pH, LDH, glucose, and protein) was assessed using Spearman’s rank correlation coefficient (CC) (with *P* < 0.05 used to define statistical significance). Multivariable binomial logistic regression was used to compare clinical outcomes to biochemical markers. The accuracy of suPAR and other conventional markers as diagnostic tests was assessed using standard sensitivity, specificity, positive likelihood ratios (PLRs), negative likelihood ratios (NLRs), and area under the curve (AUC) statistics with 95% confidence intervals (CIs). DeLong’s test was performed to compare the differences in AUCs. Statistical analysis was performed using SPSS 24.0 statistical software and receiver operating characteristic curve graphs were generated using RStudio 3.6.1 (R Foundation for Statistical Computing).

## Results

Between 2009 and 2016, 93 patients presenting to our center with pleural effusions secondary to infection (excluding frank pus) were recruited and had biological samples stored. As control subjects, 31 cases of malignant effusions and 16 transudative effusions were also included in this analysis. The median age of patients with parapneumonic effusions was 66 years and there was a male predominance. Full patient demographics by etiology are represented in Table 1.

**Table 1.** Patient Demographics, Baseline Biochemistry, and Pleural suPAR Levels

	Parapneumonic (*n* = *93*)	Malignant (*n* = *31*)	Transudative (*n* = *16*)
Age, yr, median (IQR)	66 (46–78)	68 (61–79)	74 (62–86)
Sex, M/F, *n* (%)	57/36 (61/39)	19/12 (61/39)	10/6 (63/37)
Serum, median (IQR)			
Neutrophils, ×10^9^/L	8.50 (6.45–12.09)	6.0 (4.46–6.94)	4.41 (2.88–5.69)
CRP, mg/L	119.0 (56.5–210.9)	29.0 (5.9–72.4)	20.5 (8.3–55.2)
Pleural fluid, median (IQR)			
pH	7.32 (7.06–7.41)	7.41 (7.32–7.47)	7.53 (7.43–7.71)
Protein, g/L	44 (36–51)	45 (32–50)	20 (13–27)
LDH, IU/L	679 (432–1,1493)	476 (309–768)	176 (137–217)
Glucose, mmol/L	5.3 (3.5–6.5)	5.5 (3.3–6.7)	7.3 (6.4)
Pleural suPAR, ng/ml (range)	36.9 (20.2–124.1) (9.1–644)	15.0 (9.4–26.7) (3.0–68.0)	12.0 (8.2–13.8) (8.2–18.3)

*Definition of abbreviations*: CRP = C-reactive protein; IQR = interquartile range; LDH = lactate dehydrogenase; suPAR = soluble urokinase plasminogen activator receptor.

### Pleural suPAR Levels in All Effusions

The median pleural suPAR of pleural effusions varied significantly by etiology, with parapneumonic effusions having significantly higher levels than malignancy and transudative effusions at baseline (*P* ≤ 0.01). Pleural suPAR was strongly correlated with the commonly used pleural fluid indicators of infection: pH (CC, −0.576; *P* < 0.01), glucose (CC, −0.632; *P* < 0.01), and LDH (CC, 0.596; *P* < 0.01), but not pleural fluid protein (CC, 0.057; *P* = 0.59) across all etiologies.

### Pleural suPAR in Parapneumonic Effusions

**Figure 1.**
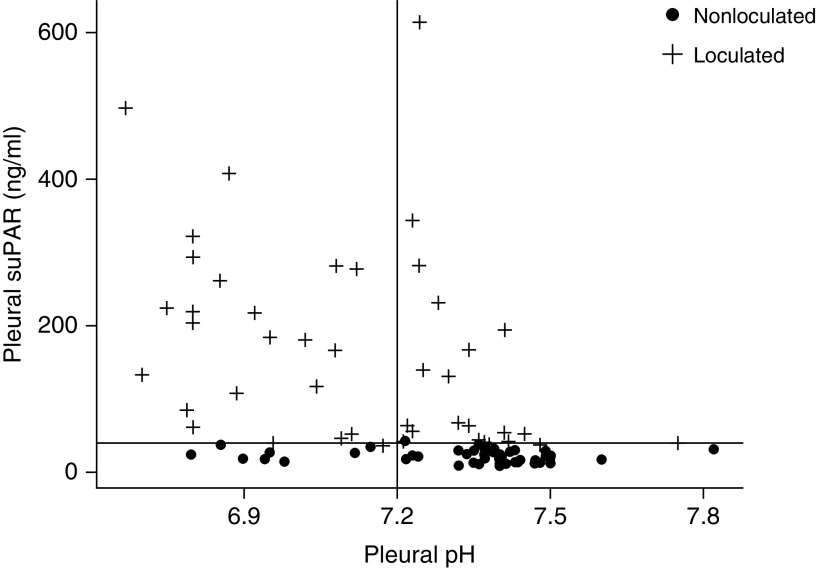
Pleural fluid pH against pleural suPAR (soluble urokinase plasminogen activator receptor) by fluid loculation (intercepts at pH = 7.2 and suPAR 35 ng/ml).

Table 2 shows the levels of pleural and serum suPAR from patients with parapneumonic effusions alongside routine pleural fluid and serum tests depending on the presence/absence of fluid loculation during hospital admission. Levels of pleural suPAR were significantly higher in loculated versus nonloculated effusions (*P* < 0.01). Using a cutoff of 35 ng/ml, pleural suPAR had a 100% sensitivity (95% CI, 91–100) for predicting pleural fluid loculations with a specificity of 91% (95% CI, 80–97), a PLR of 12.3, and an NLR of 0.0. This compared with pleural pH, which, using the conventional cutoff of 7.2, had a sensitivity of 52% (95% CI, 37–68), a specificity of 84% (95% CI, 70–93), a PLR of 3.2, and an NLR of 0.57 (Figure 1). In a multivariable analysis model, including all the analytes presented in Table 2, pleural suPAR was the only independent predictor of pleural effusion loculation during hospital admission; *see* Appendix E4.

**Table 2.** Loculated versus Nonloculated Parapneumonic Effusions and Biochemical Markers

	Nonloculated (*n* = *49*)	Loculated (*n* = *44*)	*P* Value (Univariable Analysis)
Pleural pH, median (IQR)	7.4 (7.28–7.44)	7.14 (6.88–7.33)	<0.01
Pleural protein, g/L, median (IQR)	45 (38–51)	40.0 (34.3–50.0)	0.98
Pleural LDH, IU/L, median (IQR)	516 (330–747)	1,276 (657–2,794)	<0.01
Pleural glucose, mmol/L, median (IQR)	5.7 (4.95–6.90)	3.45 (0.2–5.3)	<0.01
Pleural suPAR, ng/ml, median (IQR) (range)	22.3 (14.0–28.1) (9.1–42.3)	132.2 (52.3–229.2) (36.9–614.0)	<0.01*
Serum neutrophils, ×10^9^/L, median (IQR)	7.00 (5.51–10.32)	10.1 (7.56–13.77)	<0.01
Serum CRP, mg/L, median (IQR)	96.3 (46.0–150.3)	139.1 (75.1–247.2)	0.01
Serum suPAR, ng/ml, median (IQR) (range)	4.64 (3.66–6.41) (2.02–16.90)	6.12 (3.95–7.96) (1.94–20.9)	0.22

For definition of abbreviations, *see* Table 1.

* Significant on multivariable analysis, *see* Appendix E4.

In nine patients in whom the initial ultrasound was simple, loculations developed on subsequent pleural ultrasound and/or computed tomography scans at a median of 5 days (range, 3–10). The baseline pleural suPAR was significantly higher in parapneumonic effusions that subsequently loculated (median, 139.6 ng/ml; IQR, 41.9–312.8) compared with those that remained nonloculated (median, 22.3; IQR, 14.0–28.1) and was equivalent to effusions that were loculated from baseline (median, 131.0; IQR, 52.7–223.8) (*P*  < 0.01).

### Serum suPAR in Parapneumonic Effusions

Paired serum suPAR levels were not correlated with pleural suPAR within parapneumonic effusions (CC, 0.170; *P* = 0.11), nor any other pleural fluid marker or fluid loculation. Serum suPAR was correlated with serum CRP (CC, 0.268; *P* < 0.01) and serum neutrophils (CC, 0.233; *P* = 0.03) but not clinical outcomes.

### Pleural suPAR and Chest Tube Insertion for Parapneumonic Effusions

**Figure 2.**
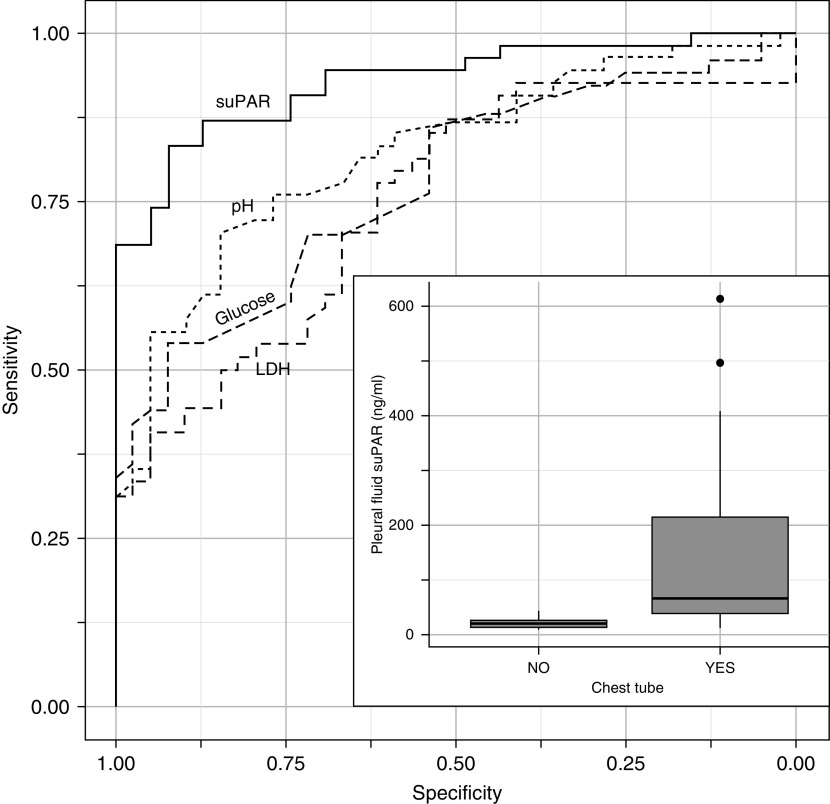
Receiver operating characteristic curves of pleural markers to predict insertion of a chest tube, plus boxplot of pleural suPAR (soluble urokinase plasminogen activator receptor) and insertion of chest tube. LDH = lactate dehydrogenase.

Of the conventional pleural fluid markers for predicting chest tube insertion (pH, glucose, and LDH), pleural pH was the most accurate (AUC, 0.82; 95% CI, 0.73–0.90; sensitivity, 54%; specificity; 95%; PLR, 10.5; and NLR, 0.5, using 7.2 as a cutoff). Pleural suPAR (alone) was superior to pleural pH (alone) at predicting the insertion of a chest tube for drainage of infected pleural effusions (AUC, 0.93; 95% CI, 0.89–0.98; *P* = 0.01, using DeLong’s test). Using a cutoff of 35ng/ml, pleural suPAR had an 83% sensitivity, a 92% specificity, a PLR of 10.8, and an NLR of 0.2 (Figure 2). In a multivariable logistic regression, pleural pH (*P* = 0.02), pleural LDH (*P* = 0.05), a neutrophilic effusion (*P* = 0.05), and pleural suPAR (*P* = 0.01) were significant indicators for chest tube insertion; *see* Appendix E4.

### Pleural suPAR and Referral for Medical/Surgical Rescue Therapies

Pleural suPAR was superior to all other conventional markers combined at predicting the need for rescue therapies (intrapleural fibrinolytics or thoracic surgery) with an AUC of 0.92 (95% CI, 0.87–0.98; *P* = 0.02, using DeLong’s test). Using a cutoff of 65 ng/ml, pleural suPAR was 94% sensitive and 84% specific (PLR, 6.0 and NLR, 0.1) at predicting the referral for these therapies (16 of the 93 patients) (Table 3).

**Table 3.** Median Pleural suPAR and Conventional Biomarker Levels by Clinical Outcomes

Biomarker Levels	Conservative Management (*n* = *39*)	Chest Tube (*n* = *54*)	Fibrinolytics and/or Surgery (*n* = *16*)
Pleural pH, median (IQR)	7.40 (7.35–7.47)	7.14 (6.89–7.35)	6.93 (6.80–7.29)
Pleural LDH, IU/L, median (IQR)	451 (317–906)	1,004 (565–2,645)	1,119 (203–4,657)
Pleural glucose, mmol/L, median (IQR)	6.2 (5.0–7.1)	4.1 (0.3–5.6)	0.6 (0.2–5.4)
Pleural suPAR, ng/ml, median (IQR)	19.7 (13.3–27.9)	65.9 (38.4–218.3)	218.7 (141.8–312.1)

*Definition of abbreviations*: IQR = interquartile range; LDH = lactate dehydrogenase; suPAR = soluble urokinase plasminogen activator receptor.

**Table 4.** Pleural pH and suPAR Levels in Malignant Effusions

	Nonloculated (*n* = *12*)	Delayed Loculation (*n* = *9*)	Loculated (*n* = *10*)
Pleural pH, median (IQR)	7.46 (7.43–7.50)	7.39 (7.32–7.44)	7.33 (7.18–7.53)
Pleural suPAR ng/ml, median (IQR)	10.7 (7.3–14.0)	17.4 (12.3–25.2)	36.5 (21.9–51.3)

*Definition of abbreviations*: IQR = interquartile range; suPAR = soluble urokinase plasminogen activator receptor.

**Figure 3.**
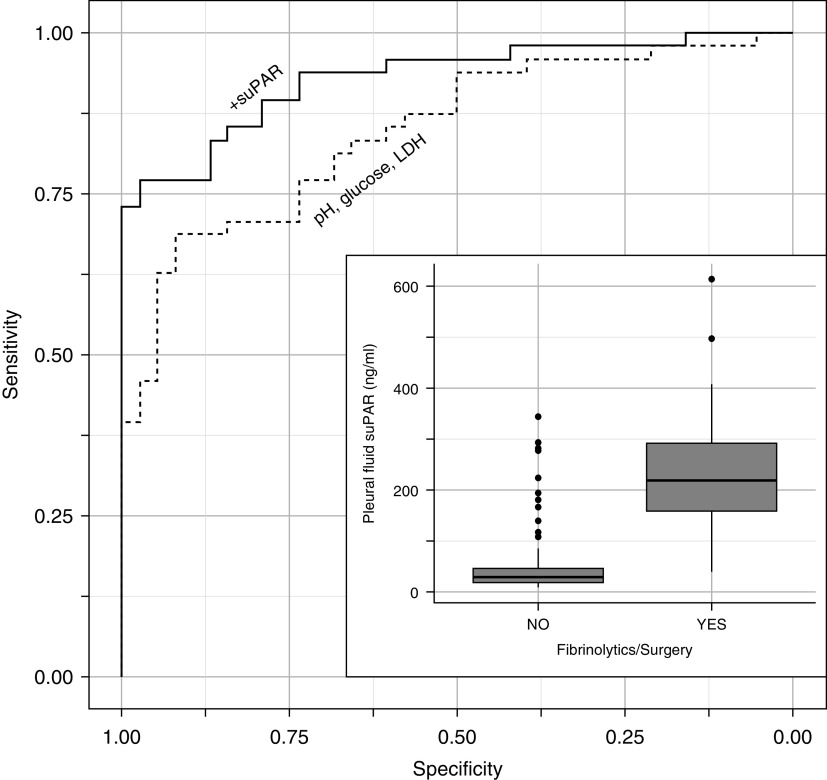
Receiver operating characteristic curves of conventional pleural biomarkers combined (pH, glucose, and LDH) and the additional benefit of pleural suPAR (soluble urokinase plasminogen activator receptor) at predicting the use of fibrinolytics/surgery, plus boxplot of pleural suPAR and use of fibrinolytics/surgery. LDH = lactate dehydrogenase.

The combination of markers that are conventionally used to define a complex parapneumonic effusion (including pleural pH < 7.2 or pleural glucose ≤ 3.0 mmol/L [≤55 mg/dl] or pleural LDH > 1000 IU/L) (4) had an AUC of 0.76 (95% CI, 0.71–0.81) for predicting rescue therapies (Figure 3). Pleural suPAR was the only significant baseline predictor of rescue therapies (*P* = 0.01); *see* Appendix E4.

### Pleural suPAR in Malignant Effusions

**Figure 4.**
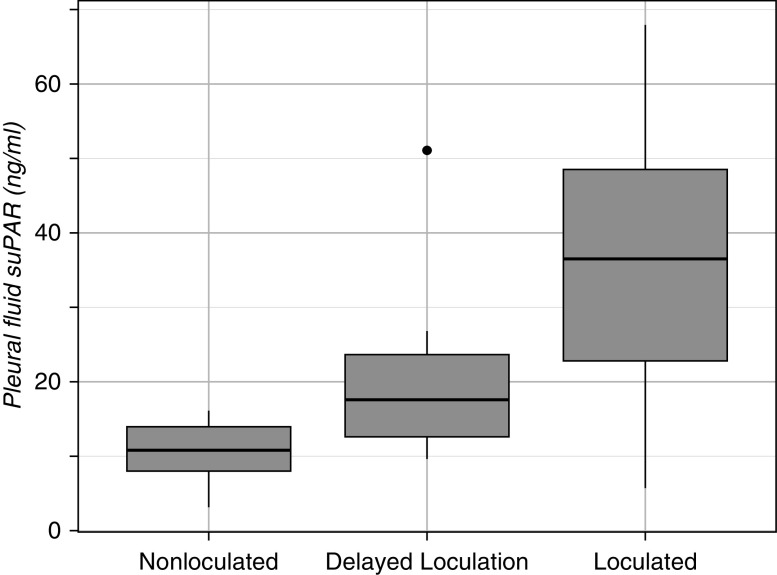
Boxplot of pleural suPAR (soluble urokinase plasminogen activator receptor) levels in malignant effusions.

Pleural suPAR levels were significantly higher in malignant effusions that were loculated at the time of pleural fluid analysis (*P* < 0.01) (Table 4). We performed a further analysis to assess whether baseline pleural suPAR levels could predict future malignant loculations. The “delayed loculation” group included effusions that started out nonloculated (simple) and became loculated (over a period of 4–6 mo). Baseline pleural suPAR levels were nonsignificantly higher in the delayed loculation group compared with those that remained nonloculated (*P* = 0.19) (Figure 4).

## Discussion

In this prospectively recruited cohort of patients presenting with parapneumonic effusions, high pleural suPAR could predict the insertion of a chest tube with an AUC of 0.93 (95% CI, 0.89–0.98). It could predict the presence or development of loculations with considerable accuracy. A high pleural suPAR was also indicative of the referral for intrapleural fibrinolytics and/or thoracic surgery and was superior to conventional pleural fluid biomarkers.

The optimal management of parapneumonic effusions is contentious and the topic of several ongoing research studies. Much of the uncertainty relates to the difficulty of predicting which patients require formal drainage of their effusion and which will resolve with conservative management (antibiotics) alone. Guidelines recommend formal drainage in the case of frank pus or a positive Gram stain/culture of pleural fluid (3, 4). Given low culture rates of fluid from complex parapneumonic effusions (14), formal drainage is also recommended if the pleural fluid pH is less than 7.2. This threshold was first suggested by Light and colleagues in 1980 after a case series of 90 patients showed that low pH effusions (*n* = 10) tended to need chest tube drainage (2). In 1995, Heffner and colleagues performed an elegant meta-analysis of the studies relating to the topic of using pleural pH, glucose, or LDH in distinguishing complicated and uncomplicated parapneumonic effusions (5). From the seven included studies (251 patients) they concluded that pleural pH was the best performing analyte at a cutoff of 7.21. However, they also recognized that given the observational nature of the seven studies and the fact that this analyte had become “entrenched in clinical practice,” it required “validation in well-designed prospective studies.” pH falls due to lactic acid and $\dot{V}$co_2_ by bacteria within the pleural space (15, 16). Although indicating the presence of bacteria it is prone to both false positives and negatives in the need for invasive pleural management, it has never been prospectively validated in this regard (17).

A crucial factor in the management of parapneumonic effusions is the development of pleural thickening, septations, and loculations. The tendency for loculation development is not only associated with more severe infection; it also reduces the likely success of simple fluid drainage versus the need for more invasive medical (e.g., intrapleural fibrinolytics) or surgical therapies. Again, a low pleural pH is more likely in loculated effusions (18) but is inaccurate because it is a sequalae of numerous biochemical reactions, not simply the derangement of normal fibrinolysis (19), so cannot be used as an indicator for fibrinolytics or surgery. Other markers to predict which patients might require more invasive management of their parapneumonic effusion have been elusive. Pleural fluid biomarkers such as procalcitonin (20), CRP (21), and calprotectin (22) have been tested in parapneumonic effusions but, given these markers focus on neutrophilic activation and/or a general increase in chemo-cytokine activity, they are no more specific than pH in prognostication. Recent studies have tested cytokines involved in the production of pleural fluid (23) but fewer have focused on those related to loculation development, for which suPAR seems a more specific target.

**Figure 5.**
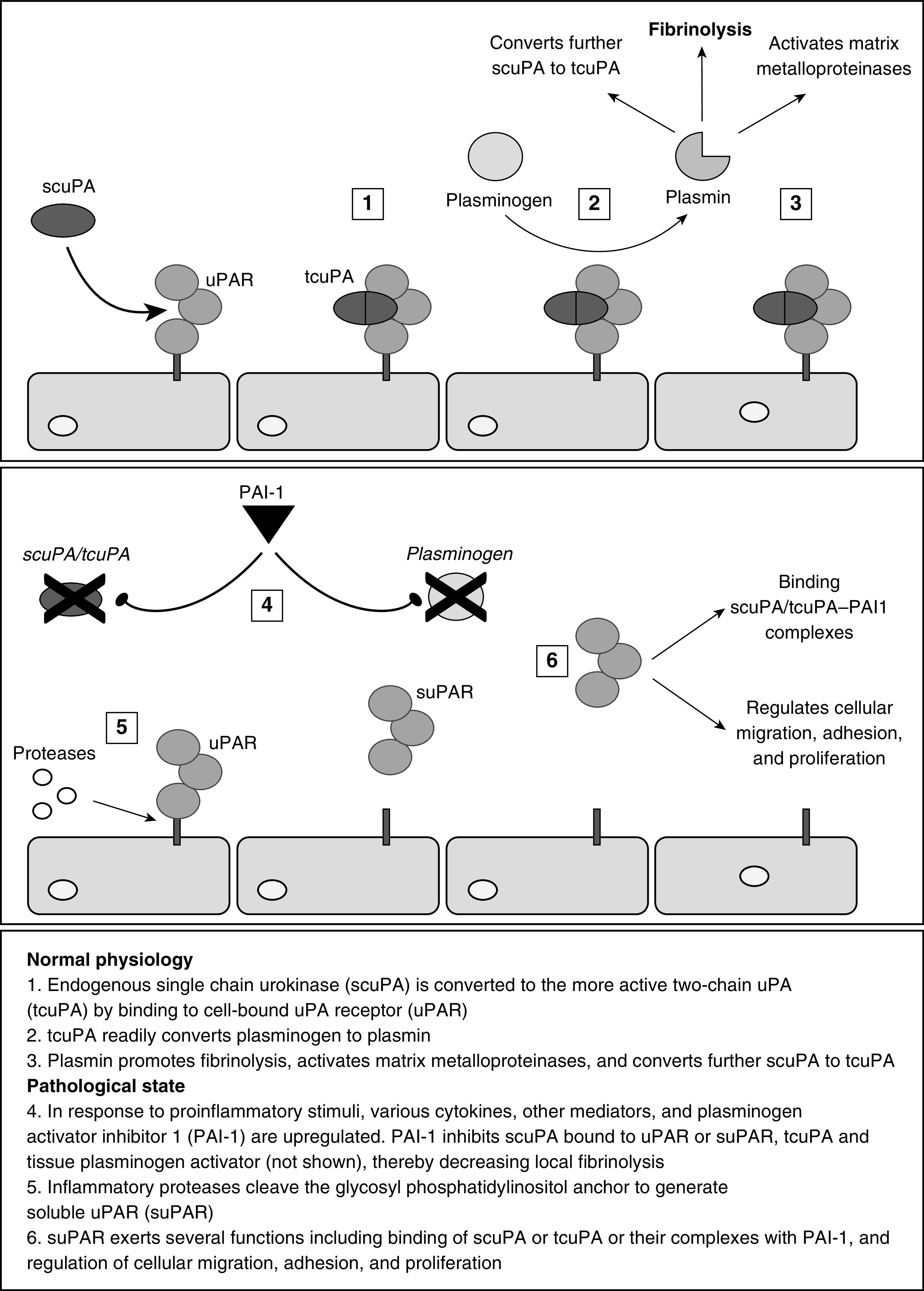
The biology of suPAR and the urokinase-type plasminogen activator system.

Loculations develop due to derangement of the normal fibrinolysis cascade mediated by the uPA system (Figure 5). This is composed of a proteinase called uPA, a cell-bound uPAR, and suPAR. suPAR, the soluble form of uPAR, is a glycoprotein with a molecular weight of 55 to 60 kDa. uPAR is cleaved from its glycosylphosphatidylinositol anchor by various proteases related to infection and inflammation. The uPA system is involved in pericellular proteolysis, cell migration, and tissue remodeling. Most notably, uPA, once bound to uPAR, catalyzes the conversion of plasminogen into plasmin, a potent endogenous fibrinolytic. It has been demonstrated in both animal models and humans that the development of pleural loculations is related to levels of PAI-1, which is released by pleural mesothelial cells (24). PAI-1 inhibits uPA and, therefore, the conversion of plasminogen, as well as suppressing the activity of several other endogenous and therapeutic fibrinolytics. Given these pathological roles, it is logical that PAI-1 itself could serve as a biomarker of pleural organization; however, this is limited by the instability and variation of the enzyme (25). We have shown that in parapneumonic effusions pleural suPAR is dramatically raised in the presence or even future development of pleural loculations. The biological role of suPAR in pleural fluid are less well understood. Although neutrophil-bound uPAR is inversely correlated with suPAR levels in critically ill patients (26), binding of scuPA to suPAR increases its plasminogen activation activity, suggesting that suPAR can augment pleural fluid plasminogen activator activity and plasmin generation (27). This interaction could localize plasminogen activation within pleural fluid, similar to that which occurs at cell surfaces (28). Measurement of suPAR could potentially provide a method to assess the capacity of pleural fluids to support uPA-related plasminogen activator activity. The MIST-2 (Multicenter Intrapleural Sepsis Trial 2) trial demonstrated that the combination of intrapleural alteplase and DNase improved radiographic appearance and reduced hospital length of stay and surgical referral rates in pleural infection (29). However, uncertainty persists around patient selection and optimal timing of both fibrinolytics and surgical intervention given the difficulty of predicting the course of pleural infection at baseline. Given its ability to predict the development of complicated effusions, suPAR may be an opportunity to use biomarkers in lung precision medicine to identify which patients are likely to require admission for drainage and early rescue treatments, addressing a “specific clinical unmet need” (30).

The higher levels of pleural suPAR in loculated effusions of a malignant etiology compared with nonloculated suggest that malignant locule development follows a similar fibrinogenesis cascade. Levels were lower in loculated malignant effusions compared with loculated parapneumonic effusions, suggesting the process is more subacute in malignancy. However, there were several cases in which malignant effusions had pleural suPAR levels similar to loculated parapneumonic effusions, limiting its utility as a diagnostic test in the sometimes challenging clinical situation of distinguishing an advanced malignant effusion from infection. We also tested the ability of pleural suPAR to predict the development of loculations within malignant effusions that were simple at baseline. Levels were nonsignificantly higher in the delayed loculation group and, given the small numbers involved, this relationship needs further investigation.

This study has some limitations that may affect the generalizability of its findings. Although suPAR levels were done en bloc and, therefore, researchers were blind to the results, the other biochemical results (pH, LDH, and glucose) were part of clinical care so would have affected a physician’s management. This is a weakness of all research that has attempted to study the true utility of pleural pH and may actually strengthen the conclusions of the suPAR results. The decision to insert a chest tube is influenced by many different biochemical and radiological factors and may vary according to the treating physician. Despite this, pleural suPAR was the most accurate baseline variable (including all biochemical and radiological markers) at identifying patients who went on to have a chest tube or rescue therapies. Second, some of the clinical outcomes such as fibrinolytic use and surgical referral may have been confounded by other factors not related to the pleural infection alone. The recently presented PILOT (Pleural Infection Longitudinal Outcome Study) trial has demonstrated that a significant proportion of patients with the most serious pleural infection do not go on to have surgery due to frailty and/or comorbidity (31). Additionally, the routine use of the fibrinolytic agents tPA (tissue plasminogen activator)/DNase was not adopted for several years into this study’s recruitment. Both factors may explain why many patients with a high pleural suPAR did not have fibrinolytics or surgery, although the biomarker was highly specific for these rescue therapies. Third, suPAR levels were measured on clinical samples that had been frozen for up to 10 years (median 6 yr). However, serum suPAR levels have been shown to be resistant to up to eight freeze–thaw cycles and stable over a 5-year period, limiting the impact on this analysis (32, 33). Finally, the urgent nature of pleural infection treatment means that any biomarker should be able to be analyzed rapidly. This study used the commercial suPARnostic ELISA, which would not fulfill this requirement. However, more rapid analytical platforms are available, including a suPARnostic Quick Triage point-of-care device or turbidimetric assay (suPARnostic TurbiLatex).

### Conclusions

The management of parapneumonic effusions has been dictated by crude measures of inflammation and bacterial replication for decades. The uPA system plays a key role in the development of pleural loculations and is a theoretically promising target of study. This prospective cohort study demonstrated that high pleural fluid suPAR levels are strongly correlated with the development of loculations in parapneumonic effusions, as well as subsequent invasive management, including chest tube drainage, fibrinolytics, and thoracic surgery. A comprehensive assessment of the utility of pleural suPAR in parapneumonic effusions requires a prospective multicenter trial of suPAR-guided management versus standard care.

## Supplementary Material

Supplements

Author disclosures
