# Supplementary material for: Pleural Fluid suPAR Levels Predict the Need for Invasive Management in Parapneumonic Effusions
Source: Am J Respir Crit Care Med. 2020 Jun 15;201(12):1545–53. doi: 10.1164/rccm.201911-2169OC (PMC7301729; doi:10.1164/rccm.201911-2169OC)
Supplement: Supplements [file rccm.201911-2169OC.html]

Pleural Fluid suPAR Levels Predict the Need for Invasive Management in Parapneumonic Effusions | American Journal of Respiratory and Critical Care Medicine

- arnold\_data\_supplement.pdf (914 KB)
- disclosures.pdf (229 KB)
