# Supplementary material for: Pleural Fluid suPAR Levels Predict the Need for Invasive Management in Parapneumonic Effusions
Source: Am J Respir Crit Care Med. 2020 Jun 15;201(12):1545–53. doi: 10.1164/rccm.201911-2169OC (PMC7301729; doi:10.1164/rccm.201911-2169OC)
Supplement: Supplements [file rccm.201911-2169OC_arnold_data_supplement.pdf]

Pleural Fluid suPAR Levels Predict the Need for Invasive Management in Parapneumonic Effusions

David T Arnold, Fergus W Hamilton, Karen T Elvers, Stuart W Frankland, Natalie Zahan-Evans,

Sonia Patole, Andrew Medford, Rahul Bhatnagar, and Nicholas A Maskell

ONLINE DATA SUPPLEMENT

Appendix E1; Standardised Diagnostic criteria for pleural effusions (North Bristol Pleural Investigation Study (08/H0102/11)). Online Data Supplement.

Malignant
Malignant pleural fluid cytology or biopsy
or
Histologically confirmed pulmonary/extra-thoracic malignancy with radiographic evidence of metastasis to ipsilateral pleura on CT.
or
Radiological changes meeting Leung's criteria which have progressed in keeping with malignancy on interval CT scan in the correct clinical context (1).
or
Autopsy confirming pleural malignancy

Empyema
Clinical presentation suggestive of sepsis/infection
And (one or more of the following)
a/. Pleural fluid gram stain or culture positive
or
b/. Frank pus on pleural aspiration

Complicated parapneumonic effusion (CPE)
Clinical presentation suggestive of sepsis/infection (and follow up for at least 6 months inconsistent with pleural malignancy)
And (one or more of the following)
a/. Pleural fluid pH ≤7.2
or
b/. Pleural fluid glucose ⩽ 3.0 mmol/L
or
b/. Pleural fluid LDH > 1000 IU/L (upper limit of serum LDH is 480 U/L)

Simple Parapneumonic effusion (SPE)
Clinical presentation suggestive of sepsis/infection with appropriate chest radiology and pleural fluid which does not fulfil the criteria for empyema or CPE (above)
And
Resolution of effusion on CXR after antibiotics

Connective tissue disease (including RA)
Systemic features or known diagnosis of connective tissue disease
And
chest radiology (including CT imaging) showing benign features (eg doesn't meet any of Leung's criteria) with at least 6 months follow-up and /or pleural biopsy negative for malignancy.

Pulmonary embolism
Evidence of PE on CTPA
And
No alternative explanation for pleural effusion on cross sectional imaging or pleural fluid analysis. (where the CT shows no evidence of pleural thickening – which would suggest another cause)

BAPE or diffuse pleural thickening due to asbestos
History of asbestos exposure or evidence of pleural plaques on CT
And
a/. Stable or improving CT appearances with follow-up for at least 12 months.(The development of enfolded lung is allowed)
or
b/. Negative thoracoscopy (benign pleural biopsy)

Congestive Cardiac Failure (CCF)
History and examination features of CCF
or
Evidence of at least moderate LV systolic or diastolic failure or severe valvular disease on echo
or
Improvement of effusion and symptoms with diuretic therapy

Coronary artery bypass graft (CABG) effusion
CABG in 3 months prior to development of pleural effusion in the absence of an alternative cause

Hepatic hydrothorax
Known history or clinical presentation consistent with liver disease
And
Recurrent transudative pleural effusion
And
Negative cytology

Renal failure or hypoalbuminaemia
Biochemical confirmation of renal failure or hypoalbuminaemia in the absence of clinical, radiological or pleural fluid analysis suspicious of an alternative cause.

TB pleuritis
Culture or Acid-alcohol-fast-bacilli (AAFB) positive sputum, pleural fluid or pleural tissue
And
Resolution of pleural effusion with anti TB therapy at 6 month follow-up.

Inflammatory pleuritis (Non-specific pleuritis)

Demonstration of non-specific inflammatory pleuritis on pleural biopsy

And

Presentation not in keeping with parapneumonic effusion (see above)

And

Follow-up for 12 months without progression that would suggest a malignant cause.

Undiagnosed

Exhaustive investigations including 12 months follow-up with interval CT scans has not demonstrated a diagnosis

or

Patient unfit for further investigation and follow up

or

Patient died without definitive diagnosis and no post mortem examination conducted

1. Leung, A.N., N.L. Muller, and R.R. Miller, CT in differential diagnosis of diffuse pleural disease. AJR Am J Roentgenol, 1990. 154(3): p. 487-92.

Appendix E2. Parapneumonic effusion management

Indication for chest tube insertion for parapneumonic effusions

Chest tube insertion should be considered for the following indications;

Clinical presentation consistent with parapneumonic effusion AND fulfilling at least one of the following criteria;

- Purulent pleural fluid
- Pleural fluid pH ≤ 7.2
- Pleural fluid glucose ≤ 3.0 mmol/L
- Pleural fluid lactate dehydrogenase (LDH) > 1000 IU/L
- Pleural fluid gram stain and/or culture positive for bacteria

If, despite not meeting the above criteria, the patient fails to respond to initial medical therapy with ongoing markers of infection/sepsis then chest tube drainage should be reconsidered.

Indication for intrapleural fibrinolytics

Intrapleural fibrinolytics (tPA 5mg and DNase 5mg delivered via the chest tube) should be considered after a minimum of 24-48hours chest tube drainage.

Assuming no contraindications to treatment, intrapleural fibrinolytics should be considered when:

- Thoracic ultrasound imaging has revealed pleural fluid septation or loculation which is felt to be clinically important
- The chest tube has stopped draining but there remains a clinically important residual effusion on chest radiograph/CT scan/pleural ultrasound
- The patient continues to show clinical or biochemical signs of infection despite good chest tube drainage and appropriate antibiotic therapy

Indication for thoracic surgical referral

Referral to the thoracic surgeons, if appropriate, should be considered if:

- intrapleural fibrinolytics have failed to result in clinical improvement
- intrapleural fibrinolytics are contraindicated in the context of failed chest tube drainage (above criteria)
- the degree of fluid complexity precludes the use of chest drainage or fibrinolytic therapy

Appendix E3. Sample handing protocol and additional experiments on the effect of sample collection tubes and centrifugation.

Sample handling protocol for main analysis

Pleural fluid samples

- Pleural fluid collected at diagnostic thoracentesis into plain (non-heparinized/non-citrated) collection tubes
- Pleural fluid centrifuged for 20minutes at 1000G
- Supernatant pipetted into 1.5ml Eppendorf tubes (pellet discarded)
- Samples stored at -70oC for future analysis or immediately processed.

Blood samples

- Blood samples collected by venepuncture at the time of diagnostic thoracentesis into serum separator gel bottle (yellow-top).
- Blood sample left to rest upright for 30 mins
- Blood sample centrifuged for 20minutes at 1000G
- Following centrifugation, the liquid supernatant (serum) is transferred into 1.5ml Eppendorf tubes
- Samples stored at -70oC for future analysis or immediately processed.

Impact of collection tube type on suPAR results

A subset of 8 patients had pleural fluid and blood collected using plain (gold top serum separator tubes for blood), sodium-citrate (blue) and EDTA (lavender) tubes.

Blood samples were handled as per tube manufacturers protocol;

https://www.thermofisher.com/uk/en/home/references/protocols/cell-and-tissue-analysis/elisa-protocol/elisa-sample-preparation-protocols/plasma-and-serum-preparation.html

Pleural fluid samples were spun at 1000G for 20minutes as per our protocol shown above and the supernatant collected and frozen at -70oc prior to *en-bloc* analysis.

For both pleural fluid and blood samples suPAR levels were analysed using the suPARnostic ELISA in duplicate.

Figure A&B; Line graphs of suPAR level depending on sample collection tube. A-Pleural fluid, B- Blood

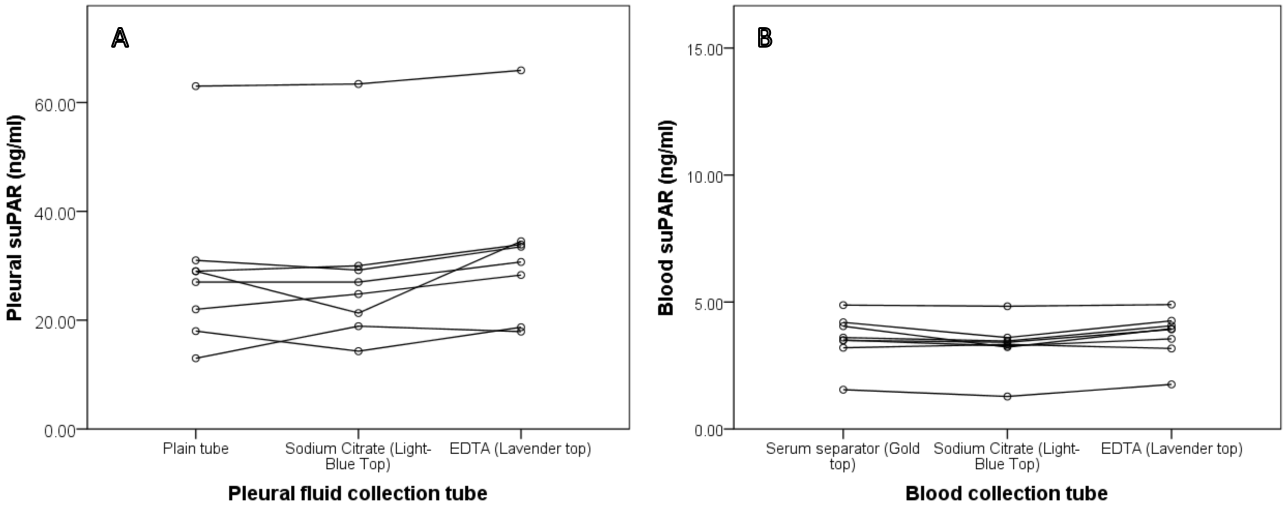

For pleural fluid there was slight variation in pleural suPAR levels with a slight increase in levels using EDTA tubes.

For blood samples there was no significant variation in suPAR levels depending on the collection tube type, see Figure B.

Impact of centrifuge speed on pleural suPAR results

To test the impact of varying centrifuge speed on pleural suPAR levels, 3 patients with parapneumonic effusions had pleural fluid collected at diagnosis. Pleural fluid was collectd in plain tubes (as per the protocol for the main analysis) and centrifuged at 3 different speeds for 20 minutes before analysis (as well as a tube that was not spun at all). The supernatant was collected and anlaysed using the suPARnostic ELISA in duplicate.

There was no significant difference between the samples but there was a trend towards increasing suPAR levels as the centriuge spin was increased, see Figure C.

Figure C; Boxplot of pleural suPAR levels for 3 patients depending on centrifuge speed at sample collection.

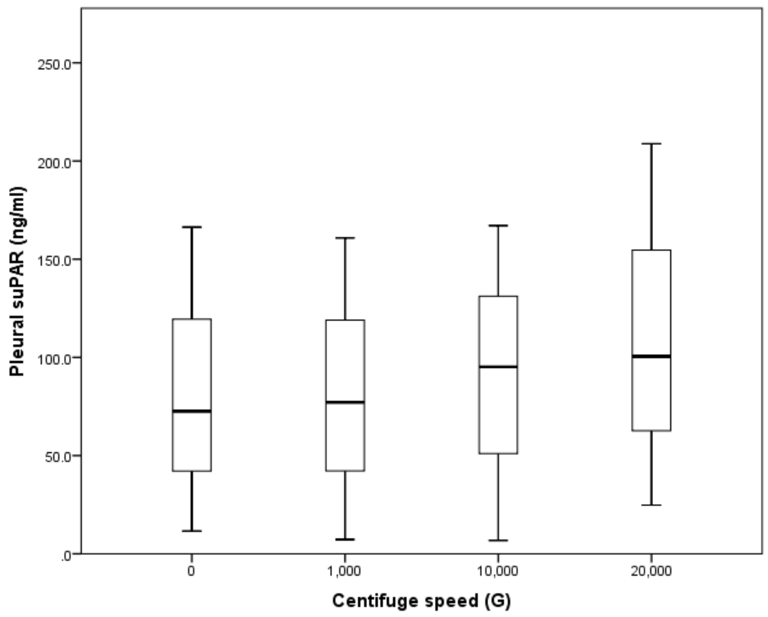

Appendix E4. Multivariable binomial logistic regression tables

Baseline predictors of parapneumonic effusion loculation development (n=93)

Factor	Regression Estimate	Std. Error	z value	p-value
Pleural suPAR	0.5688	0.2763	2.059	0.0395
Pleural Glucose	-0.0531	0.4815	-0.110	0.9122
Pleural protein	-0.2707	0.2093	-1.293	0.1959
Pleural LDH	-0.0001	0.0016	-0.076	0.9397
Pleural pH	1.9691	4.3523	0.453	0.6509
Serum Neutrophils	-0.3383	0.5496	-0.616	0.5381
Serum CRP	0.0022	0.0165	0.131	0.8956
Serum suPAR	0.2745	0.2894	0.949	0.3427
suPAR- soluble urokinase Plasminogen Activator Receptor, LDH- lactate dehydrogenase, CRP- C reactive protein				

Baseline predictors of chest tube insertion in parapneumonic effusions (n=93)

Factor	Regression Estimate	Std. Error	z value	p-value
Pleural pH	-6.4282	2.6101	-2.463	0.0138
Pleural Protein	0.04990	0.0600	0.831	0.4061
Pleural LDH	-0.0025	0.0012	-2.005	0.0450
Pleural Glucose	-0.1338	0.1888	-0.709	0.4786
Neutrophilic effusion*	4.3911	2.2373	1.963	0.0497
Pleural suPAR	0.2965	0.1214	2.442	0.0146
Serum neutrophils	-0.2042	0.1768	-1.155	0.2482
Serum CRP	0.0121	0.0076	1.581	0.1139
Serum suPAR	0.2023	0.2743	0.737	0.4609
Effusion size on chest radiograph (over 50% of hemithorax)	2.3041	1.4473	1.592	0.1114
Loculation on baseline ultrasound	2.7099	2.0081	1.349	0.1772
suPAR- soluble urokinase Plasminogen Activator Receptor, LDH- lactate dehydrogenase, CRP- C reactive protein, * Defined as >50% neutrophils on pleural differential cell count				

Baseline predictors of rescue therapy (fibrinolytics or surgery) in parapneumonic effusions (n=93)

**Factor**	**Regression Estimate**	**Std. Error**	**z value**	**p-value**
Pleural pH	-1.2451	3.1952	-0.390	0.6968
Pleural Protein	0.1028	0.0669	1.537	0.1243
Pleural LDH	0.0001	0.0001	0.449	0.6537
Pleural Glucose	-0.1338	0.2618	-0.511	0.6094
Neutrophilic effusion*	-4.0691	-2.4064	-1.691	0.0908
Pleural suPAR	0.0157	0.0062	2.540	0.0111
Serum neutrophils (x10^9)	0.0230	0.0796	0.289	0.7727
Serum CRP	0.0074	0.0075	0.979	0.3278
Serum suPAR	0.1810	0.1958	0.924	0.3552
Effusion size on chest radiograph (over 50% of hemithorax)	0.5352	1.0530	0.508	0.6113
Loculation on baseline ultrasound	2.7061	1.7652	1.533	0.1252
suPAR- soluble urokinase Plasminogen Activator Receptor, LDH- lactate dehydrogenase, CRP- C reactive protein, * Defined as >50% neutrophils on pleural differential cell count				
